# Antibacterial and Antiviral Properties of *Pinus densiflora* Essential Oil

**DOI:** 10.3390/foods12234279

**Published:** 2023-11-27

**Authors:** Yu Jin Oh, Yeong-Su Kim, Jae Woo Kim, Dae Wook Kim

**Affiliations:** Department of Bioindustrial Research, Baekdudaegan National Arboretum, Bonghwa-gun 36209, Republic of Korea; oyj0705@koagi.or.kr (Y.J.O.); yskim@koagi.or.kr (Y.-S.K.); kjww2000@koagi.or.kr (J.W.K.)

**Keywords:** antibacterial, antiviral, *Pinus densiflora*, pine trunk oil, gas chromatography–mass spectrometry, essential oil, fungi, microassay, herbal medicine, functional food

## Abstract

The Korean mountains are home to the Korean red pine (*Pinus densiflora*). Pine needle oil has been used as a food additive and a traditional herbal medicine; however, any health-related properties of its trunk oil remain unknown. Herein, we assessed antibacterial and antiviral properties of essential oil extracted from the trunk of *P. densiflora*. Th extracted oil was hydrodistilled using a Clevenger apparatus and analyzed using gas chromatography–mass spectrometry. The antimicrobial activity of the oil was tested using the microbroth dilution technique against 10 bacterial species (6 g-positive and 4 g-negative) and fungi. The extract exerted strong antimicrobial activity against *Vibrio parahaemolyticus*, *Bacillus cereus*, *Listeria monocytogenes*, *Propionibacterium acnes*, and *Malassezia furfur* (minimum inhibitory concentration = 10 mL/L). Additionally, it exhibited dose-dependent activity against influenza virus A and feline coronavirus. Furthermore, among 20 identified constituents accounting for 98.7% of the oil contents, the major components included 3-cyclohexene-1-methanol (10.12%), 2-(4-methylcyclohexyl)-2-propanol (9.09%), fenchone (8.14%), *O*-isopropyltoluene (6.35%), and isothymol methyl ether (6.14%). The *P. densiflora* trunk essential oil showed antibacterial and antiviral activities that depended on its chemical composition and the microbial strains tested herein. The essential oil can be used as an antimicrobial agent and disinfectant.

## 1. Introduction

The Pinus genus of the Pinaceae family consists of 115 species of coniferous trees commonly known as pines. In particular, *Pinus densiflora* Siebold & Zuccarini is the dominant species in South Korean forests, accounting for approximately 25% of the total forest area in the country [1]. The species, also known as Korean red pine, is distributed across East Asia, including Korea, Japan, China, and the Russian Far East [2].

Certain pine species are timber species of substantial commercial value. The bark, cones, pollen, and needles of these trees serve as primary sources of various traditional medicines and health foods globally [3]. Furthermore, numerous scientific studies on pine tree extracts have revealed diverse bioactive compounds with pharmacological potential, including antioxidant, antimicrobial, anti-inflammatory, antidiabetic, anticancer, and anti-aging properties [4,5,6,7]. The flavor compounds found in pine needle tea exhibit biological activity and show potential for effectively treating conditions such as bronchial asthma, arteriosclerosis, and inflammation. Therefore, pine needle powder, tea, liquor, and soft drinks are commercially available as supplements or health foods [8]. Similarly, pine bark, renowned for its potent hemostatic, anti-inflammatory, and analgesic properties, has been commonly used in traditional remedies [9]. In Korea, pine needles hold a place in traditional folk remedies and, historically, have been consumed as a food source during times of famine, as they were highly valued for their affordability and the presence of bioactive substances [10]. Specifically, pine needle extracts are rich in antioxidants, offering potential anticancer effects, aiding in heavy metal detoxification, and showing antibacterial and anti-inflammatory properties [11]. Indeed, owing to their antioxidant effects, pine extracts have extensive applications in various products, including soaps, essential oils (EOs), hangover relief agents, and health drinks [12]. Moreover, the Korean Ministry of Food and Drug Safety has formally acknowledged pine needle distillate–concentrate as a functional ingredient in health functional foods. The statement “May aid in maintaining healthy blood glucose levels” is deemed acceptable for inclusion when utilizing pine needle distillate–concentrate, and the product has received approval for consumption as a health functional food.

EOs derived from aromatic plants are classified as naturally occurring secondary metabolites. They consist of volatile mixtures typically in an oily form. Further, EO components have been extensively evaluated for their safety [13] and are known for their diverse biological effects, including antioxidant, antifungal, insecticidal, and antibacterial activities [14]. EOs have a range of industrial applications, including pharmaceuticals, perfumery, cosmetics, food and beverages, soap, fumigants, and detergents [15]. Moreover, to date, over 3000 EOs have been identified, with approximately 300 being commercially used in the food and pharmaceutical industries [16]. Particularly, plants within Pinaceae are noteworthy for containing a significant concentration of EOs, primarily composed of volatile aromatic terpenes, including hydrocarbons, and oxygenated derivatives such as monoterpenes and sesquiterpenes [17,18,19]. Hence, EOs extracted from plants within Pinaceae have various applications, including aromatherapy, [16] owing to their anti-inflammatory [20], antioxidant [21], and antimicrobial [11,22] activities. Specifically, collectively known as phytoncide, EOs derived from pine needles show strong antibacterial properties against a variety of Gram-negative and -positive bacterial strains. In contrast, to date, our knowledge of the chemical composition and biological activities of pine trunk EOs remains limited. Therefore, in this study, we aimed to evaluate the antibacterial and antiviral properties of EO extracted from the trunk of *P. densiflora*.

## 2. Materials and Methods

### 2.1. EO Extraction from P. densiflora Siebold & Zuccarini (PDEO)

Trunk tissue of *P. densiflora* Siebold & Zuccarini was collected in July 2021 from Bonghwagun, Gyeongsangbuk-do, Korea, and the species was authenticated by D. H. Lee of Baekdudaegan National Arboretum, Korea. A 300 g sample of air-dried wood chips was finely ground and subjected to hydrodistillation at 100 °C for 2 h in a Clevenger-type apparatus (NEOS, Milestone, Milan, Italy); 2 L of distilled water was added. The volatile oil was dried over anhydrous sodium sulfate, filtered through a 0.45 μm membrane disk filter, and stored in a sealed vial at 4 °C until use. The yield of the hydrodistillate from pine trunks was 0.36% (*w*/*w*).

### 2.2. Determination of Antibacterial and Antifungal Activity

#### 2.2.1. Screening for Antimicrobial Activity

Antimicrobial activity of PDEO was assessed against a panel of microorganisms, including Gram-positive and -negative bacterial strains and fungi obtained from the American Type Culture Collection (ATCC; Rockville, MD, USA). Gram-positive bacteria included *Bacillus cereus*, *Clostridium perfringens*, *Staphylococcus aureus*, *Listeria monocytogenes*, *Streptococcus mutans*, and *Propionibacterium acnes*, and Gram-negative bacteria included *Escherichia coli, Salmonella typhi*, and *Vibrio parahaemolyticus*, grown in TSB broth at 37 °C for 24 h. The fungal strain tested was *Malassezia furfur* (for ATCC numbers, see Table 1). *Streptococcus aureus* and *S. epidermidis* were grown in TSB broth, and *M. furfur* was maintained in brain heart infusion (BHI) medium in a shaking incubator at 32 °C for 48 h. Ampicillin and ketoconazole were used as a quality control for bacteria and fungi.

#### 2.2.2. Microdilution Assay

To determine the minimum inhibitory concentration (MIC), a serial dilution bioassay was conducted. Bacterial inocula were cultured for 24 h in Wilkins-Chalgren anaerobic broth (10^8^ CFU/mL, 0.5 McFarland’s standard). A 1.0 mL/mL stock solution of PDEO was prepared in RPMI 1640 medium for each tested sample (HyClone, Logan, UT, USA). This PDEO stock solution was serially diluted 5- to 10-fold with RPMI 1640 medium, resulting in final oil concentrations of 500.00, 100.00, 10.00, 1.00, 0.10, and 0.01 μL/mL. Subsequently, the tested samples in these different concentrations were evaluated in 96-well plates for antibacterial activity. Briefly, 20 μL of each tested sample was placed in a well, and 180 μL of bacterial suspension was added to each well. The positive control comprised 180 μL of RPMI medium and 20 μL of bacterial suspension, whereas the negative control contained 200 μL of RPMI medium. Then, the micro plates were incubated at 37 °C for 24 h, and subsequently, 10 μL of a 2 mg/mL aqueous solution of p-iodonitrotetrazolium violet (Sigma Aldrich, Saint Louis, MO, USA) was added to each well prior to further incubation at 37 °C for 24 h for bacteria and 48 h for fungi. MIC was defined as the lowest concentration resulting in inhibition of the color change in p-iodonitrotetrazolium violet measured using a microplate reader (SpectraMax iD3 Multi-Mode Microplate Reader, Molecular Devices, San Jose, CA, USA), which implied this was the lowest concentration at which no visible microbial growth was detected. Each treatment group was replicated twice.

### 2.3. Determination of Antiviral Activity

#### 2.3.1. Cell Culture and Viruses

Crandell-Rees feline kidney (CRFK) cells purchased from ATCC (Manassas, VA, USA) were grown in Dulbecco’s minimum essential medium (DMEM, HyClone, Logan, UT, USA) supplemented with 10% fetal bovine serum (FBS, Gibco), 100 U/mL penicillin, and 100 mg/mL streptomycin (GIBCO-BRL, Grand Island, NY, USA) and maintained in a 5% CO_2_ incubator at 37 °C. The feline coronavirus (FCoV), FIPV WSU 79-1146 (FIPV1146), was acquired from ATCC (Manassas, VA, USA). FCoV FIPV1146 was originally isolated from the liver, spleen, and lungs of a 4-day-old male Persian kitten from a case of neonatal death [23].

#### 2.3.2. Cytotoxicity Assay

EO cytotoxicity was evaluated using the cell proliferation reagent WST-1 (Roche, Rotkreuz, Switzerland) according to manufacturer instructions. PDEO was diluted in dimethylsulfoxide to a concentration of 1 to 10^−7^% before being introduced to the growing medium at the desired concentration (1 to 10^−7^%).

#### 2.3.3. FIPV-79-1146 Inoculum for In Vitro CRFK Infection Study

CRFK cells were cultured in 96-well plates to 80% confluency, at which point the medium was changed to DMEM without serum immediately prior to infection. PDEO was serially diluted 10-fold with 1 to 10^−7^%, and the mock treatment (medium only) was added to the wells, then the cells were immediately inoculated with FCoV FIPV1146 at a multiplicity of infection value of 0.1. CRFK cells developed visible cytopathic effects (CPEs) characterized by multiple regional cell fusion and syncytium formation. Infected cells were then incubated at 37 °C for 16–20 h until CPEs appeared. The supernatant of each well was collected and frozen at −80 °C for virus titer determination. Briefly, CRFK cells were seeded in 96-well plates and infected with 10-fold dilutions of the thawed supernatants collected earlier. Each sample was analyzed in eight replicates. The plates were incubated for 72–96 h until no further CPE was observed. Plates were then incubated for 2 h and stained with crystal violet; each well was scored for CPEs. TCID_50_ was calculated using the Reed–Muench method [24].

### 2.4. Determination of Antiviral Activity (H1N1)

#### 2.4.1. Cell Culture and Viruses

Madin–Darby canine kidney (MDCK) cells were obtained from ATCC (Manassas, VA, USA). The RAW 264.7 cells were cultured in DMEM (HyClone, Logan, UT, USA) supplemented with 10% heat-inactivated FBS (HyClone, Logan, UT, USA), 100 μg/mL streptomycin, 100 U/mL penicillin, and 2 mM l-glutamine. The cells were cultivated in a 10 cm^2^ culture dish and incubated in a humidified atmosphere containing 5% CO_2_ at 37 °C and sub-cultured every 2 days. Influenza virus A/PR/8/34 (H1N1) obtained from the ATCC was used and stored at −80 °C.

#### 2.4.2. Plaque Assay for Influenza A (H1N1) Virus Infectivity

The infectivity of the influenza A (H1N1) virus on MDCK cells was assessed using a plaque assay. For the virus pretreatment with the inhibitor (PDED), the inhibitor and the virus (6.477 log_10_ plaque-forming unit (PFU)/mL) were incubated at a 1:9 ratio at room temperature for 1 h. The viral suspensions were then serially diluted 10-fold in DMEM medium and inoculated onto confluent cell monolayers at 37 °C in 5% CO_2_ for 1 h. After virus adsorption, the inocula were aspirated, and 1 mL of 1.5% low-melting-point agarose overlay, prepared in culture medium containing 2 μg/mL of trypsin, was added to each well. Following incubation at 37 °C in 5% CO_2_ for 48–72 h, the cell monolayer was fixed with 4% formaldehyde for 1 h. Subsequently, the agarose overlay was removed, and the cell layer was stained with 0.5% crystal violet. Assays were conducted in 6-well plates in triplicate. Plaques were then counted, with sterilized distilled water serving as the untreated control. Data shown represent a measure of PFU reduction (log_10_ PFU/mL).

### 2.5. Chromatographic Analysis

Gas chromatography–mass spectrometry (GC-MS) analysis was performed using a Shimadzu GC-MS/MS TQ8050 NX (Shimadzu, Kyoto, Japan) instrument equipped with a split injector and a flame ionization detector to separate and detect the constituents of PDEO. Analytes were separated with an Agilent 60 m × 0.25 mm ID (df = 0.25 µm) DB-5MS capillary column (Folsom, CA, USA). The flow of the helium carrier gas was measured at 1.0 mL/min. The oven was maintained at 50 °C for 5 min under isothermal conditions. Subsequently, the temperature was programmed to increase to 280 °C at a rate of 2 °C/min and maintained at that point for 20 min. The temperature of the injector was recorded as 270 °C. The diluted sample (1 μL, diluted at a ratio of 100:1 in acetone) was injected into the system using a split ratio of 1:2. The linear velocity of the helium carrier gas was 24.4 cm s^−1^ at 30 °C and a split ratio of 1:50. The capillary column and temperature settings used for GC-MS detection were consistent with those previously outlined for GC analysis. The temperature of the ion source was recorded at 270 °C. The temperature of the interface was maintained at 270 °C, while the mass spectra were acquired using an energy of 70 eV. The sector mass analyzer was configured to perform a scan ranging from 35 to 550 atomic mass units (amu) at a frequency of 0.2 s. Chemical constituents were identified by comparing the retention time and molecular weight of the detected components with the mass spectra properties of the known components available in the Willey libraries and National Institute of Standards and technology library.

### 2.6. Statistical Analysis

All assays were performed in triplicate. The arithmetic mean ± standard error of the mean (SEM) of control and experimental results were compared using Student’s *t*-test. Statistical significance was set at *p* < 0.05.

## 3. Results

### 3.1. Antimicrobial and Antifungal Activity

The antimicrobial and antifungal activities of PDEO were evaluated using broth microdilution assays. The EO exerted varying levels of growth inhibition on the selected microbial strains, demonstrating variation in susceptibility among different microbial species (Table 1). The MICs observed were within 10–500 μL/mL. The lowest activity was observed on the Gram-negative bacteria Salmonella typhi and the Gram-positive bacteria Streptococcus mutans at a MIC of 500 μL/mL. In contrast, Gram-negative V. parahaemolyticus; Gram-positive B. cereus, L. monocytogenes, and P. acnes; and the fungus *M. furfur* showed the lowest MIC (i.e., 10 μL/mL), representing the highest antimicrobial activities. Gram-negative *E. coli* and Gram-positive *C. perfringens* and *S. aureus* displayed similar levels of resistance at a MIC of 100 μL/mL. These findings indicate that the EO obtained via hydrodistillation has significant antibacterial and antifungal properties against a wide range of human pathogenic microorganisms.

### 3.2. Cell Viability

**Figure 1:**
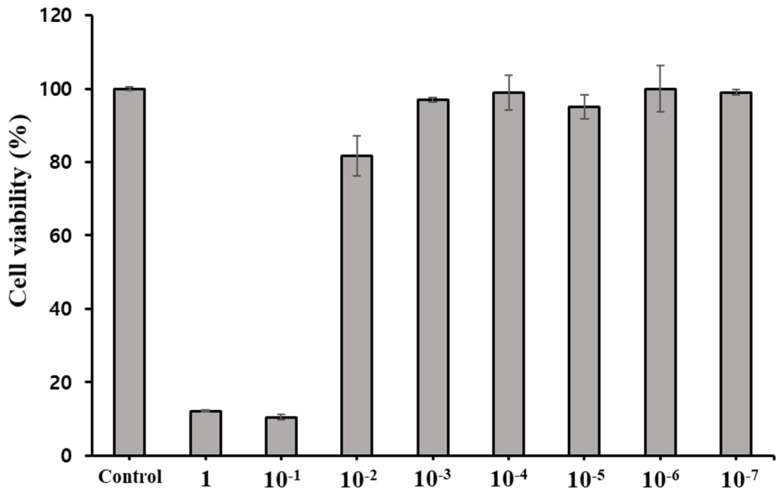
Viability of Crandell−Rees feline kidney cells treated for 2 h with *P. densiflora* Siebold & Zuccarini essential oil at different concentrations. The results are shown for non-treated cells (negative control), defined as 100%. Each value represents the mean ± SEM of eight experiments in triplicate.

Cytotoxicity of PDEO was assessed with the cell proliferation reagent WST-1 (Roche, Rotkreuz, Switzerland). The PDEO solution was appropriately diluted to a concentration of 0.001% using dimethyl sulfoxide. Subsequently, this diluted solution was introduced into the growth medium to achieve the desired final concentration range of 1 to 10^−7^% (Figure 1).

### 3.3. Antiviral Activity (FCoV)

**Figure 2:**
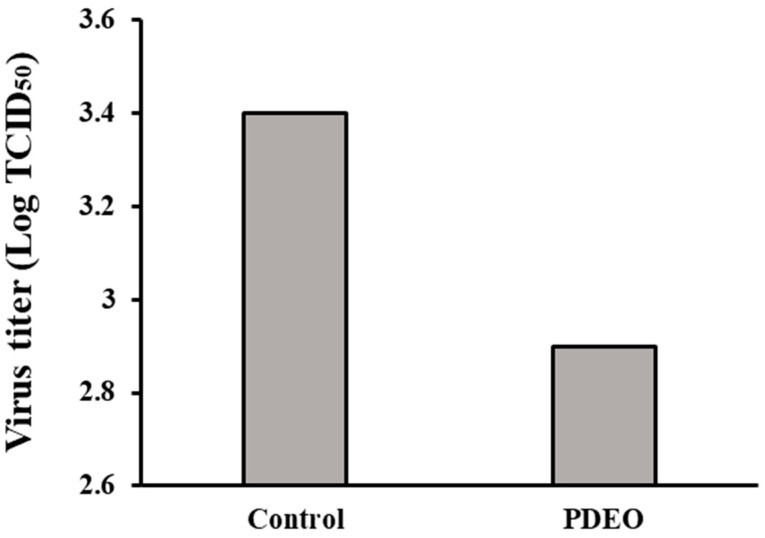
Antiviral activity of *P. densiflora* Siebold & Zuccarini essential oils (PDEOs) in combination Cells were infected with FCoV and simultaneously treated with PDEO for 2 h at 25 ± 1 °C. The results are shown for non-treated cells (negative control), defined as 100%. Viral titers were evaluated using the endpoint dilution method, expressed as log_10_ TCID_50_, and plotted against TEO at different concentrations.

Our results demonstrated the ability of PDEO to inhibit viral infection. We determined the TCID_50_ values of PDEO and demonstrated FCoV inhibition in CRFK cells. The FCoV virus batch had an output titer of 10^3.4^ TCID_50_/mL. Immediately after treatment, the titers of the remaining viruses on PDEO treatment declined by approximately 0.5 log_10_ TCID_50_ (Figure 2).

### 3.4. Antiviral Activity (H1N1)

**Figure 3:**
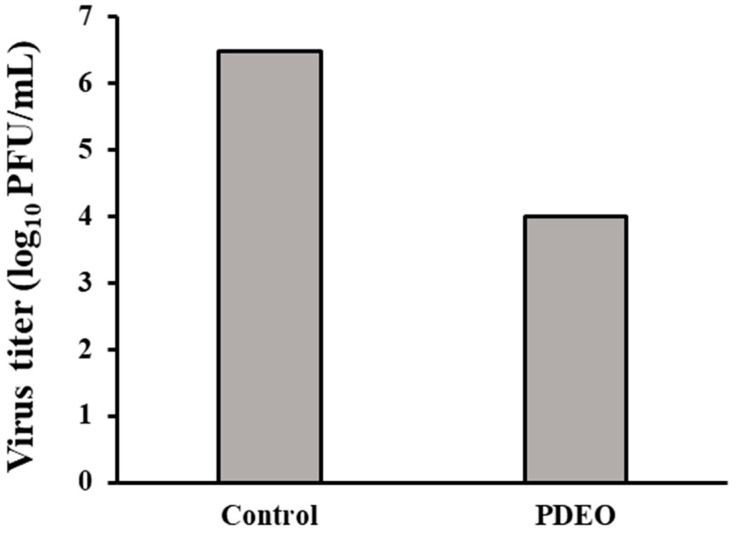
Antiviral activity of the combined *P. densiflora* Siebold & Zuccarini essential oils (PDEOs). Cells were infected with the H1N1 virus and simultaneously treated with PDEO for 2 h at 25 ± 1 °C. After infection, the cells were washed and overlaid with agarose at 37 °C for 48 h. The results are shown for non-treated cells (negative control), defined as 100%. Within treatments, PDEO caused a significant decrease in the plaque-forming unit (PFU) (log_10_PFU/mL) compared with that in the untreated influenza virus group.

Our results showed the ability of PDEO to inhibit viral infection. The plaque assays showed an approximate 99.67% reduction in H1N1 viral production upon treatment with PDEO (Figure 3). During pretreatment, PDEO showed inhibitory effects against H1N1. PDEO showed 2.477 log inhibition. The PFU (log_10_ PFU/mL) was decreased compared to that in the non-treated influenza virus group.

### 3.5. Chemical Composition of PDEO

**Figure 4:**
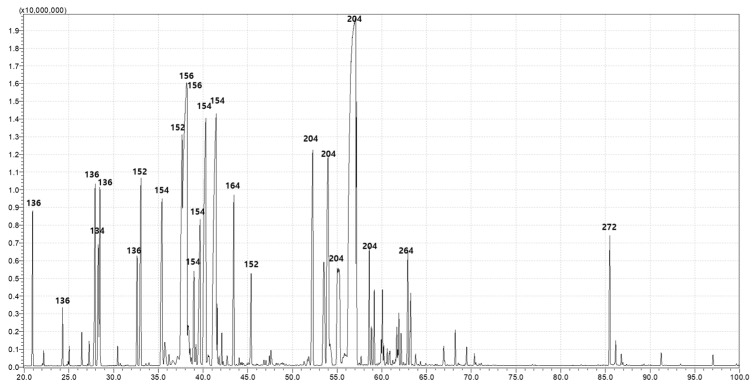
*Pinus densiflora* Siebold & Zuccarini essential oil constituents were identified using gas chromatography and gas chromatography–mass spectrometry (GC-MS).

The GC-MS showed that PDEO is composed of 7 major (≥5.0%) and 13 minor constituents, as revealed by the comparison of mass spectral data (Figure 4 and Table 2). The seven major constituents were the following: 3-cyclohexene-1-methanol, p-menth-1-en-4-ol, 2-(4-methylcyclohexyl)-2-propanol, fenchone, (-)-borneol, *O*-isopropyltoluene, and isothymol methyl ether (10.12, 9.87, 9.09, 8.14, 7.83, 6.35, and 6.14%, respectively).

## 4. Discussion

Thousands of foodborne pathogenic bacteria and 250 types of foodborne diseases which affect the health and safety of humans and animals have been identified [25]. Among these bacteria, *Campylobacter*, *Salmonella*, *L. monocytogenes*, *Escherichia* spp., *C. perfringens*, *S. aureus*, *B. cereus*, and *V. parahaemolyticus* are major foodborne pathogenic bacteria responsible for most foodborne illnesses, including recurrent intestinal infections, central nervous system disorders, arthritis, renal disorders, and blindness [26,27,28,29,30,31,32]. *Streptococcus mutans* is a Gram-positive, facultative anaerobic bacterium with the ability to produce acid and thrive in acidic environments [33,34]. The presence of this microbe within dental biofilms plays a critical role in the initiation and progression of cariogenic dental plaques [35]. In turn, *P. acnes* is another Gram-positive anaerobic bacterium that lacks motility. It colonizes the skin and hair follicles, thriving in sebaceous environments in which it uses sebum as a primary source of nutrients [36]. Furthermore, the involvement of sebum in the development of acne has been established [37] and is reportedly due to the release of lipases, proteases, and hydrolases by *P. acnes* into sebum, which subsequently leads to the promotion of oxidative stress, inflammation, and tissue damage [38]. Similarly, *M. furfur* is a lipophilic yeast that constitutes a component of the indigenous human cutaneous microbiota. The colonization of skin surfaces by *M. furfur* has been linked to a range of dermatological conditions, including atopic dermatitis and dandruff [39,40].

EOs are secondary metabolites known to inhibit or delay the growth of bacteria, yeasts, and molds [41,42,43]. Their specific antimicrobial properties are contingent upon their chemical composition and determine a series of responses that impact the entirety of the bacterial cells. EOs from the trunk of *P. densiflora* Siebold & Zuccarini have a fresh forest scent and range in color from colorless to light yellow. These oils have traditionally been used to flavor or preserve foods, as well as to instill fragrances in cosmetics and aromatherapy. Gas chromatography analysis of EOs identified 20 constituents, including 3-cyclohexene-1-methanol, 2-(4-methylcyclohexyl)-2-propanol, fenchone, *O*-isopropyltoluene, and isothymol methyl ether. Several studies have revealed that the chemical composition of essential oils varies depending on the pine species. Needle essential oil from *P. halepnsis* contained 23 compounds, the most abundant of which were α-pinene and α-myrcene. *P. bruntia* oil contained 21 compounds, the most abundant of which were α- and β-pinene. There were 19 compounds found in *P. pinaster* oil, the most abundant of which were α-caryophyllene and α-pinene. And only 12 compounds were found in *P. pinea* oil, with α-pinene being the most abundant [44]. A crucial characteristic of EOs and their components is hydrophobicity, which enables them to disrupt the lipids of the bacterial cell membrane and mitochondria, thus increasing bacterial cell membrane permeability [45,46]. Most EOs exhibit a more potent effect on Gram-positive than on Gram-negative bacteria, likely due to differences in cell membrane composition [41,47,48]. Consistently, our study showed that Gram-positive bacteria, such as *B. cereus* (ATCC 14579), *L*. *monocytogenes* (ATCC 15313), and *P. acnes* (ATCC 11828), were more susceptible to EOs than Gram-negative bacteria, such as *E. coli* (ATCC 25922) and *S. typhi* (ATCC 6539). Mutlu-Ingok et al. [49] examined the antibacterial activity of steam-distilled *Elettaria cardamomum* (cardamom) and *Cuminum cyminum* (cumin) EOs against *E. coli* ATCC 25922 (MIC = 3.75 µL/mL, and 7.50 µL/mL, respectively) and *S. aureus* ATCC 9144 (MIC = 7.50 µL/mL, and 7.50 µL/mL, respectively) using broth microdilution. Similarly, Pesavento et al. [50] showed the antibacterial potential of *Salvia officinalis* EOs against *L. monocytogenes* ATCC (MIC of 60 µL/mL) and *S. aureus* ATCC (MIC = 12.5 µL/mL), also using broth microdilution. However, the antimicrobial activities of *P. densiflora* EO were somewhat weaker than those of cardamom and cumin EOs. Seemingly, the difference in antibacterial activities among the EOs may be related to the concentration and nature of the oil components, the specific oil composition, functional groups, and structural configuration of the components and their possible synergistic interactions. Therefore, we determined the in vitro antimicrobial activity of commercial EOs against foodborne pathogens and food-spoilage bacteria to identify potential candidates for use in food preservation.

Seasonal influenza poses a substantial public health burden annually, causing numerous upper respiratory tract infections. Influenza viruses are highly contagious, spreading easily through airborne droplets and leading to rapid transmission during seasonal epidemics, resulting in numerous deaths worldwide due to severe complications. In April 2009, a novel pandemic influenza A (H1N1) virus emerged in Mexico, quickly spreading globally and causing high morbidity and mortality [51]. Despite the development of effective antiviral agents, such as amantadine and oseltamivir, there is a significant risk of emergence of resistant viruses and the associated side effects [52]. Therefore, safe and effective antiviral drugs for therapeutic or prophylactic purposes are urgently needed. Consequently, numerous research groups have focused on developing new and effective antiviral drugs, particularly from natural resources [53,54,55,56].

Setzer et al. [33] examined the in vitro antiviral activity of commercially available EOs, including those of thyme (*Thymus vulgaris*), bergamot (*Citrus bergamia*), lemongrass (*Cymbopogon flexuosus*), cinnamon (*Cinnamomum zeylanicum*), and lavender (*Lavandula angustifolia*), against influenza type A (H1N1). These oils were assessed both in liquid form at a concentration of 0.3% and in vapor form. In the liquid phase, thyme, bergamot, lemongrass, and cinnamon oils demonstrated complete inhibition (100%) of H1N1, whereas lavender essential oil showed an 85% inhibition. However, in the vapor phase, only cinnamon leaf essential oil achieved 100% inhibition after 30 min of exposure. Thyme, bergamot, lemongrass, and lavender EOs showed inhibition rates of 70%, 95%, 90%, and 80%, respectively. Therefore, in this study, the antiviral activity of PDEO was considered highly effective, as it caused 99.67% inhibition of the H1N1 virus at 2 h after exposure to PDEO.

Coronaviruses (CoVs), of the *Coronaviridae* virus family, are enveloped, single-stranded, positive-sense RNA viruses that infect a wide range of animal hosts. Currently, CoVs are classified into four genera: alphacoronavirus, betacoronavirus, gammacoronavirus, and deltacoronavirus. These viruses are known to cause diarrhea in cattle and pigs, as well as upper respiratory diseases in chickens [57]. The feline coronavirus (FCoV), a member of the alphacoronavirus genus, specifically infects cats. There are two distinct types of FCoVs, namely type I FCoV (FCoV-I) and type II FCoV (FCoV-II), with the latter being derived by recombination between FCoV-I and canine CoV (CCoV) [58].

This study provided evidence supporting the hypothesis that PDEO inhibits the infectivity of pandemic influenza A (H1N1). Thus, PDEO has the potential to be a safe and abundantly available treatment for the prevention of influenza infections. Furthermore, we demonstrated that PDEO exerted antiviral activity against FCoV in CRFK cells, opening several avenues for the potential application and therapeutic possibilities of PDEO against human and animal coronaviruses.

## 5. Conclusions

Gram-positive bacteria such as *B. cereus*, *C. perfringens*, *S. aureus*, *L. monocytogenes*, and *P. acnes* had their rates of multiplication slowed down by the essential oils that were extracted from the trunks of *P. densiflora* trees. Gram-negative bacteria, such as *E. coli*, *S. typhi*, and *V. parahaemolyticus*, experienced the greatest retardation in their rate of growth as a result of their presence. The anti-influenza activity of PDEO was tested using a plaque assay against the influenza virus strain A/PR/8/34 (H1N1); this assay also showed that PDEO inhibited FCoV in CRFK cells. In general, our findings lead us to the conclusion that the essential oil (EO) extracted from the trunk of *P. densiflora* possesses antibacterial and antiviral activities.

## Figures and Tables

**Table 1:** Antimicrobial activity of *P. densiflora* Siebold & Zuccarini essential oils against Gram-negative and -positive bacteria and fungi.

Microorganism	Strain	MIC		
		*P. densiflora* Siebold & Zuccarini Essential Oil (μL/mL)	Ampicillin (μg/mL)	Ketoconazole (μg/mL)
Gram-negative bacteria	*Escherichia coli* ATCC 25922	100	1.0	NT *
	*Salmonella typhi* ATCC 6539	500	0.5	NT
	*Vibrio parahaemolyticus* ATCC 17802	10	10	NT
Gram-positive bacteria	*Bacillus cereus* ATCC 14579	10	0.1	NT
	*Clostridium perfringens* ATCC 13124	100	0.5	NT
	*Staphylococcus aureus* ATCC 12228	100	0.2	NT
	*Listeria monocytogenes* ATCC 15313	10	0.5	NT
	*Streptococcus mutans* ATCC 25175	500	0.2	NT
	*Propionibacterium acnes* ATCC 11828	10	0.2	NT
Fungi	*Malassezia furfur* ATCC 14521	10	NT	0.1

* NT: Not tested; MIC: Minimum inhibition concentration; ATCC: American Type Culture Collection.

**Table 2:** Chemical constituents of *P. densiflora* Siebold & Zuccarini essential oils identified using gas chromatography and gas chromatography–mass spectrometry (GC-MS).

Peak No.	Compound	RT ^a^ (min)	% Area
1	alpha-Pinene	20.94	2.87
2	beta-Pinene	24.29	0.94
3	alpha-Phellandrene	26.44	0.59
4	*O*-Isopropyltoluene *	27.92	6.35
5	beta-Phellandrene	28.46	4.18
6	(+)-4-Carene	32.61	1.48
7	Fenchone *	33.05	8.14
8	Fenchol	35.41	4.44
9	Camphor	37.65	4.31
10	2-(4-Methylcyclohexyl)-2-propanol *	38.15	9.09
11	cis-Dihydro-alpha-terpineol	38.97	4.81
12	(-)-Borneol *	39.67	7.83
13	p-Menth-1-en-4-ol *	40.31	9.87
14	3-Cyclohexene-1-methanol *	41.46	10.12
15	Isothymol methyl ether *	43.44	6.14
16	Piperitone	45.38	1.87
17	alpha-Longipinene	52.27	4.98
18	(E)-beta-Famesene	58.59	1.51
19	5-Azulenemethanol	63.21	0.83
20	Thunbergen	85.49	0.69

^a^ Retention times; * Major constituent.

## Data Availability

Data are contained within the article.
